# Quantitative RT-PCR Assays for Quantification of Undesirable Mutants in the Novel Type 2 Oral Poliovirus Vaccine

**DOI:** 10.3390/vaccines10091394

**Published:** 2022-08-25

**Authors:** Hasmik Manukyan, Rahnuma Wahid, Azeem Ansari, Erman Tritama, Andrew Macadam, John Konz, Konstantin Chumakov, Majid Laassri

**Affiliations:** 1 Division of Viral Products, Center for Biologics Evaluation and Research, US Food and Drug Administration, 10903 New Hampshire Avenue, Silver Spring, MD 20993, USA; 2 Center for Vaccine Innovation and Access, PATH, Seattle, WA 98121, USA; 3 Research and Development Division, PT. Bio Farma, Bandung, West Java 40161, Indonesia; 4 National Institute for Biological Standards and Control (NIBSC), Hertfordshire EN6 3QG, UK

**Keywords:** OPV, nOPV2, quality control, mutant variants

## Abstract

Emergence of mutations is an inherent property of RNA viruses with several implications for their replication, pathogenesis, and evolutionary adaptation. Oral poliovirus vaccine (OPV), developed by Albert Sabin, is composed of live attenuated polioviruses of three serotypes that can revert to neurovirulence during replication in cell culture and in vaccine recipients. Recently, a new modified variant of Sabin 2 virus was developed by introducing changes in its genome, making it more genetically stable to prevent the reversion. The new strain was used to manufacture novel OPV2 (nOPV2), which was approved by the World Health Organization for emergency use to stop outbreaks caused by circulating vaccine-derived poliovirus (cVDPV2). Manufacture of this improved vaccine requires close attention to the genetic heterogenicity to ensure that the levels of the undesirable mutations are limited. Preliminary studies using whole-genome Illumina sequencing (NGS) identified several genomic sites where mutations tend to occur with regularity. They include VP1-I_143_T amino acid change at the secondary attenuation site; VP1-N_171_D, a substitution that modestly increases neurovirulence in mice; and VP1-E_295_K, which may reduce the immunogenicity of the nOPV2. Therefore, to ensure the molecular consistency of vaccine batches, the content of these mutants must be quantified and kept within specifications. To do this, we have developed quantitative, multiplex, one-step reverse-transcriptase polymerase chain reactions (qmosRT-PCRs) as simple methods for quantification of these mutations. Each method uses specific short TaqMan probes with different dyes for the analysis of both mutants and non-mutants in the same sample. The quantification is done using calibration curves developed using validated reference materials. To evaluate the sensitivity and the linearity of the qmosRT-PCR method, the mutant viruses were spiked in non-mutant viruses, and nOPV2 batches were used to validate the method. The spiked samples and the nOPV2 batches were analyzed by qmosRT-PCR and NGS assays. The results showed that qmosRT-PCR is sensitive enough to detect around 1% of mutants. The percentages of mutants determined by qmosRT-PCR correlate well with the results of the NGS. Further, the analysis of the nOPV2 batches showed that the results of qmosRT-PCR correlated well with the results of NGS. In conclusion, the qmosRT-PCR is a specific, sensitive, and linear method. It could be used for quality control of the nOPV2 batches.

## 1. Introduction

OPV is a live attenuated vaccine that protects well against poliomyelitis by stimulating both humoral and mucosal immunity. However, OPV is composed of three poliovirus Sabin strains that are genetically unstable and can revert to neurovirulence during replication in both cell cultures and vaccine recipients, leading to vaccine-associated paralytic poliomyelitis (VAPP) in vaccinees or their contacts, as well as the emergence of virulent circulating vaccine-derived poliovirus (cVDPV) strains that cause polio outbreaks [1].

Since, in 2015, wild poliovirus type 2 was declared eradicated by the World Health Organization (WHO), the routine use of trivalent OPV (tOPV) was stopped to reduce the risk of VAPP and cVDPV. It was replaced by bivalent OPV (bOPV), which contains only Sabin 1 and 3 viruses, supplemented with at least one dose of inactivated poliovirus vaccine (IPV) [2,3]. IPV is known to produce inadequate mucosal immunity and, thus, the vaccinees receiving bOPV with IPV are vulnerable to being infected with type 2 poliovirus [4,5,6]. Since the switch, cVDPV2 outbreaks increased significantly [7]. To stop these outbreaks, monovalent OPV2 (mOPV2) was used, but it resulted in triggering new cVDPV2 outbreaks [8,9].

The main attenuating site in Sabin strains is located in domain V of the 5′ untranslated region (UTR), in which mutations determining reversion to virulence occur (in A_481_G in the case of Sabin 2) [10,11,12,13]. The degree of attenuation is determined by the thermal stability of domain V: the stronger hairpin structure correlates with higher neurovirulence. The A_481_G reversion occurs rapidly, within a week post-vaccination [14,15,16]. For Sabin 2 virus, the initial steps of the de-attenuation also involve nucleotide changes U_398_C in domain IV of 5′UTR and the amino acid I_143_T mutation in VP1 capsid protein [13].

Recently, two genetically stable novel OPV2 (nOPV2) vaccine candidates have been developed [17,18]. Both candidates have their domain V genetically stabilized by the replacement of all weak U-G and selected strong C-G nucleotide pairs with intermediately strong U-A pairs [19], resulting in similar thermal stability and level of attenuation of domain V as in the original Sabin 2 strain. However, the reconstituted domain V is more genetically stable because it takes at least two simultaneous mutations for the base pair to be strengthened. In addition, the critically important cis-acting replicative element (cre) was relocated from the center of the genome to the 5′ UTR, protecting the reconstituted domain V from being recombined out. Finally, nOPV2 candidate 1 (nOPV2-c1) also contains D_53_N and K_38_R amino acid changes in 3D polymerase to improve replication fidelity and reduce the recombination rate, respectively [18]. In November 2020, the WHO issued an Emergency Use Listing (EUL) recommendation for the nOPV2c1. This allows the implementation of the vaccine in countries affected by cVDPV2 outbreaks, and, currently, more than 200 million doses of nOPV2 have been used, with the anticipated safety profile confirmed [20].

In addition to monitoring of the above mutations that may affect vaccine safety, it is also important to ensure that efficacy of the new vaccine is not compromised by genetic changes that can take place during virus growth in cell culture. Preliminary experiments revealed that growth in Vero cells may result in accumulation of E_295_K mutation in the capsid protein VP1, which reduces immunogenicity. Additionally, VP1-I_143_T and VP1-N_171_D, which both modestly increase neurovirulence in transgenic mice, were also observed in vaccine lots and considered important to monitor [21].

In this communication, we describe quantitative, multiplex, one-step reverse-transcriptase polymerase chain reactions (qmosRT-PCR) for detection and quantitation of VP1-I_143_T, VP1-N_171_D, and VP1-E_295_K, which may impact the neurovirulence or immunogenicity of the nOPV2.

The results demonstrate high specificity, linearity, and sensitivity from the assays, demonstrating that they are suitable for quality control of nOPV2 vaccine.

## 2. Materials and Methods

### 2.1. Plasmids and Viruses

Plasmids containing the entire genomes of nOPV2 VP1-N_171_D, and VP1-E_295_K amino acid mutants were prepared by Dr. Andrew Macadam’s group at the National Institute for Biological Standards and Control in the UK and the plasmid that contains the nOPV2 VP1-I_143_T genome was kindly provided by Professor Raul Andino from the University of California, San Francisco, CA, USA. These plasmids were used for the recovery the nOPV2c1 mutant viruses as described below. 

Monovalent bulks of nOPV2 candidate 1 (nOPV2-c1) batches nPOL 2016C, nPOL 2018C, nPOL2 B0419, and nPOL2 B0519 as well as monovalent bulks of nOPV2 candidate 2 (nOPV2-c2) batches nPOL 2038C and nPOL 2056C and drug product nOPV2-c1 batches 2060119C, 2060219C, and 2060319C were provided by P.T. Bio Farma (Indonesia) and were used for quantification of the level of the mutant variants.

### 2.2. Recovery of nOPV2-c1 Mutant Variants

nOPV2-c1 mutant viruses were recovered from the plasmids containing T7 promotor and genomes of nOPV2 VP1-I_143_T, VP1-N_171_D, and VP1-E_295_K mutant variants as described previously [5,22] with some changes. Briefly, plasmid clones of nOPV2-c1 mutants were digested with Hind III, purified with SPRI beads (1.8×), and eluted in 45 µL H_2_O. RNA transcripts were generated from the linearized plasmids using the MEGAscript™ T7 Transcription Kit (Thermo Fisher Scientific, South San Francisco, CA, USA). To remove unincorporated nucleotides and most proteins, the nOPV2-c1 -mutant transcripts were precipitated with LiCl and the pellet was washed once with 1 mL of 70% ethanol and re-centrifuged to maximize removal of unincorporated nucleotides. Then, RNA was dissolved in sterile diethylpyrocarbonate-treated water and stored at −80 °C for downstream use. The purified RNA transcripts were analyzed by electrophoresis on 1% agarose. Then, 2–3 µg of RNA transcript was added to 10^6^ HEp-2C cells and subjected to electroporation. The transfected cells were planted in 6-well plates and incubated at 34 °C and 5% CO_2_ until the appearance of a complete cytopathic effect (CPE). After the appearance of the CPE, the plate was freeze-thawed three times, and the supernatants were used to transfect a monolayer of HEp-2C cells into 25 cm^2^ flasks. The cells were incubated until the appearance of the CPE, and the supernatants were prepared as mentioned above, aliquoted, and stored at −80 °C.

The recovered nOPV2-c1 mutant viruses were sequenced with the Illumina sequencing platform as described below to confirm their nucleotide sequences. 

### 2.3. Primers and TaqMan Oligo Probes Used for nOPV2-c1 Mutant Variant Detection and Quantification

The forward and reverse primers for each of the nOPV2 VP1-I_143_T (corresponding to U_2970_C nucleotide change), VP1-N_171_D (corresponding to A_3053_G nucleotide change), and VP1-E_295_K (corresponding to G_3425_A nucleotide change) mutant variants were selected to flank the targeted mutations (Table 1). PCR amplification with these primers resulted in DNA fragments of 74, 60, and 61 nucleotides long for nOPV2 VP1-143T, VP1-171D, and VP1-295K mutants, respectively. The TaqMan oligoprobes were designed to be short (less than or equal to 13 nucleotides), with a conjugated minor groove binder (MGB) and low melting temperature, to allow discrimination of a single point mutation (Table 1). The primers were synthesized by Integrated DNA Technologies IDT (Durham, NC, USA) and TaqMan probes were synthesized by Thermo Fisher Scientific (South San Francisco, CA, USA). 

### 2.4. qmosRT-PCR Amplification

Viral RNA was extracted from nOPV2 viruses using a QIAamp viral RNA mini kit (QIAGEN, Chatsworth, CA, USA) according to the manufacturer’s protocol. The qmosRT-PCR reactions were prepared with the QuantiFast Multiplex RT-PCR Kit (QIAGEN, Valencia, CA, USA) in a final volume of 25 μL using 2 μL of viral RNA. Mutant and non-mutant TaqMan probes were used at final concentrations of 25 nM each in a mixture with the forward and reverse primers (Table 1) at concentrations of 0.8 μM each. The RNA sample of the nOPV2-c1 virus was used as positive control, and water was used as negative control. RNAs and/or plasmids containing the genomes of nOPV2-c1 mutant and non-mutant viruses were used as reference standards: reference standards, mutant, and non-mutant plasmids/RNAs with known genome copy (GC) numbers per mL were used for extrapolation of GC numbers for mutants and non-mutants of the nOPV2 test samples.

The positive control and reference standard samples were run in duplicate, and the negative control and test samples were run in three repeats. The qmosRT-PCR procedure was performed using a real-time PCR System ViiA7 (Thermo Fisher Scientific, South San Francisco, CA, USA) at the following thermocycler conditions: one cycle of incubation for 20 min at 50 °C and 5 min at 95 °C, followed by 40 cycles each consisting of 15 s at 95 °C, 15 s at 50 °C, and 30 s at 60 °C.

### 2.5. Quantitative RT-PCR for Virus Genome Copy Number Determination

To quantify the genome copy (GC) number in each sample, a qmosRT-PCR was used. Briefly, the qmosRT-PCR reactions were prepared with a QuantiFast Multiplex RT-PCR Kit (QIAGEN, Valencia, CA, USA) in 96-well optical plates in a final volume of 25 μL using 2 μL of RNA of test and control samples and 2 μL of DNA-plasmid for standard references. The RNAs of nOPV2-c1 virus were used as positive controls, and water was used as negative control. Plasmid containing the genome of nOPV2-c1 mutant virus with a known GC number was used as a standard reference for extrapolation of the GC for nOPV2 mutants as test samples, and plasmid containing the genome of nOPV2-c1 non-mutant virus with a known GC number was used as standard reference for extrapolation of the GC number for nOPV2-c1 non-mutants as test samples.

All control and standard reference samples were run in duplicate, and the test samples were run in three repeats. The specific primer pairs and probes used for each virus are presented Table 1. The qmosRT-PCR procedure was performed as described above.

### 2.6. Deep Sequencing Analysis

RNA was extracted from nOPV2 viruses as described above and used for RNA library preparation. The RNA library was prepared using the TruSeq NEBNext Ultra II RNA Library Prep Kit for Illumina (New England BioLabs, Ipswich, MA, USA). Briefly, 100 ng of RNA was fragmented to generate a mean fragment distribution of 500 nt and priming was performed in one reaction using the buffer provided in the kit. The first and second strands of DNA were synthesized according to the manufacturer’s protocol. The resulting DNA fragments were subjected to end repair and ligated to Illumina paired end adaptors. The ligated products were size selected using AMPure XP beads, then amplified using eight cycles of PCR with multiplex indexed primers and purified by magnetic beads (Agencourt AMPure PCR purification system, Beckman Coulter, Brea, CA, USA). Then, the DNA libraries were analyzed for the size and quality with a 4200 Tape Station system (Agilent Technologies, Santa Clara, CA, USA) using a high-sensitivity D1000 Screen Tape. After obtaining the Qubit concentration for the libraries and the mean peak sizes from the Agilent Tape Station profile, paired-end sequencing was performed using a MiSeq System (Illumina, San Diego, CA, USA), producing 250 nt reads. The raw sequencing reads were analyzed with the specialized High-Performance Integrated Virtual Environment (HIVE) platform developed in-house [23]. The RNA sequences of nOPV2-c1 and nOPV2-c2 with GenBank accession numbers MZ245455 and MN654096, respectively, were used as genome references for HIVE alignment and mutations profiling. 

## 3. Results

### 3.1. Assay Design

The primers were selected based on the sequence of nOPV2-c1. They flanked the targeted mutations and produced 60–74 bp long amplicons depending on the mutation position (Table 1).

Short TaqMan probes were designed with the targeted mutation in the middle to discriminate between mutants and non-mutants with a single point mutation, as we showed previously that mutation in the middle of the oligonucleotide destabilizes the complex between the oligonucleotide and the template DNA [15,24]. The TaqMan probes include a minor groove binder (MGB) moiety at the 3′ end that increases the melting temperature (Tm) of the probe and stabilizes probe/target complexes, as well as a nonfluorescent quencher (NFQ) to absorb energy from the fluorescent dye label at the other end of the probe. FAM and VIC dyes are banded to the 5′ end of the probes to discriminate between mutants and non-mutants by dye, and there is no interference between these dyes. 

**Figure 1:**
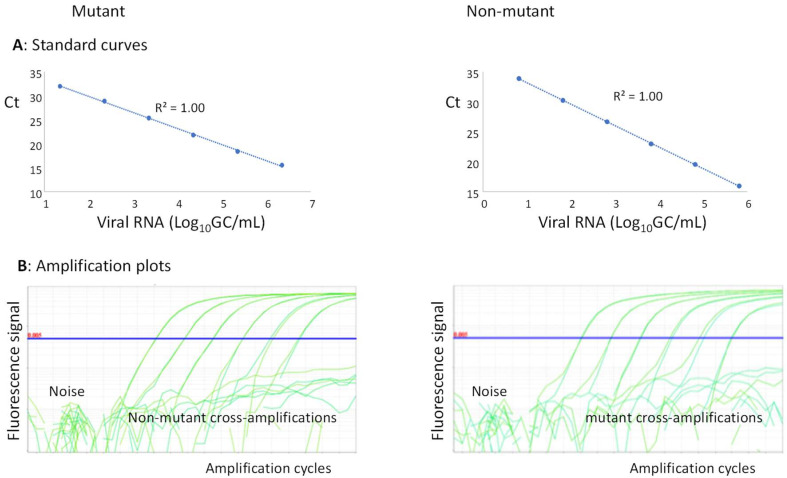
QmosRT-PCR assay: (**A**) standard curves and (**B**) amplification plots of mutants and non-mutants generated from viral RNA reference standards.

To assess the content of the mutants, a 100% mutant virus and 100% non-mutant virus were recovered from plasmids as described above. The plasmids and/or extracted RNAs from these viruses were used in each PCR run to generate standard curves for mutants and non-mutants (Figure 1A). The quantities of mutants and non-mutants extrapolated from the standard curves were used to calculate the percentages of mutants in each sample.

The baseline and the Ct-threshold could be set separately for mutants and non-mutants; the baseline was adjusted as described by the Thermo Fisher guidelines (http://surf.ed.ac.uk/wp-content/uploads/2014/02/Setting-baselines-and-thresholds-.pdf accessed on 21 August 2022), and the Ct-threshold was set wisely above the noise and higher than any cross-amplification (Figure 1B). With these settings, the method could preserve its sensitivity and was inherently specific (as the Ct-threshold was set higher than any cross-amplification between mutant and non-mutant reference standards).

### 3.2. Evaluation of the Sensitivity and Linearity and Comparison between Mutant Variant Quantifications Using qmosRT-PCR and NGS

**Figure 2:**
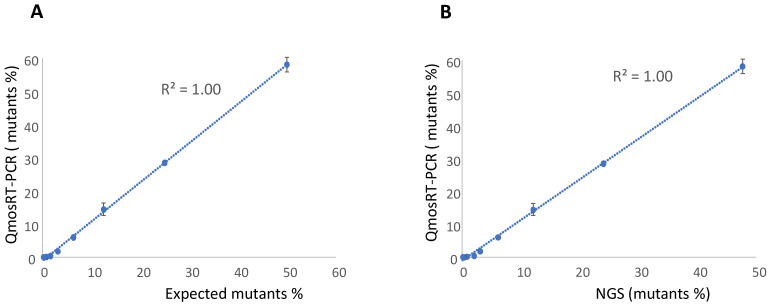
Results of the qmosRT-PCR assay using plasmid references for quantification of nOPV2-c1_171 mutants generated from the plasmid-spiked samples (mutant plasmid spiked in non-mutant plasmid): (**A**) Expected mutant percentages versus percentages generated by qmosRT-PCR and (**B**) mutant percentages generated by NGS versus those generated by qmosRT-PCR. The correlation line is drawn for the averages of the qmosRT-PCR results.

To evaluate the linearity and sensitivity of the quantification of the VP1-N_171_D mutants in nOPV2 batches with the qmosRT-PCR assay using plasmids as reference standards, we spiked plasmid that contained the mutation with non-mutant plasmid based on their genome copy (GC) numbers. The resulting percentages of mutants were 50, 25, 12.5, 6.3, 3.1, 1.6, 0.8, 0.4, 0.2, and 0.1. The spiked samples were subjected to the qmosRT-PCR assay and Illumina sequencing (NGS) as described above, and the results for the mutant percentages generated by both methods are presented in Table 2. Both methods were able to detect less than 1% of mutants, although the PCR was more accurate for spike fractions of 3% or higher (Table 2). An excellent correlation was noted between the expected results and the results of the NGS and qmosRT-PCR methods, with R^2^ = 1.00 (Figure 2A,B).

**Figure 3:**
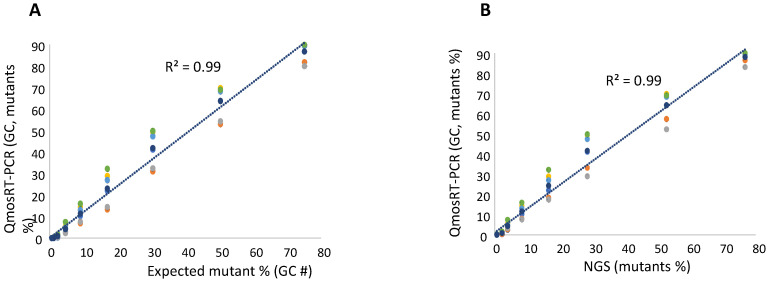
Results of the qmosRT-PCR assay using plasmid reference standards for quantification of nOPV2-c1_171 mutants, generated from the virus-spiked samples: (**A**) the mutant percentages generated by qmosRT-PCR are plotted against the expected percentages of mutants and (**B**) the mutant percentages generated by qmosRT-PCR are plotted against the results of Illumina sequencing (NGS). The correlation line is drawn for the averages of the qmosRT-PCR results. The results of different qmosRT-PCR run (Table 3) are presented with different colors.

**Figure 4:**
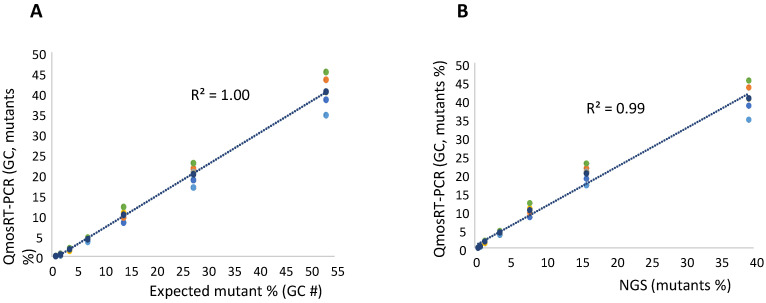
Results of qmosRT-PCR assay using plasmid refence standards for quantification of nOPV2-c1_295 mutants, generated from the virus-spiked samples: (**A**) the mutant percentages generated by qmosRT-PCR are plotted against the expected percentages of mutants and (**B**) the mutant percentages generated by qmosRT-PCR are plotted against the results of Illumina sequencing (NGS). The correlation line is drawn for the averages of the qmosRT-PCR results. The results of different qmosRT-PCR runs (Table 4) are presented with different colors.

**Figure 5:**
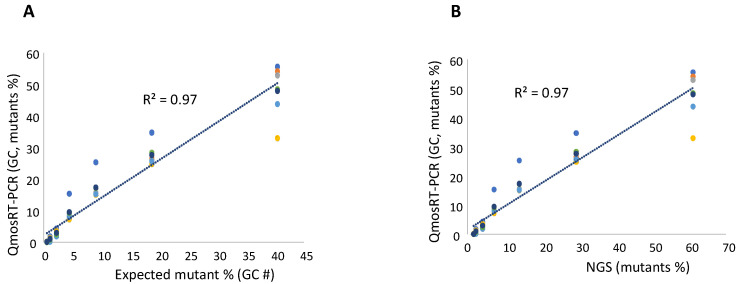
Results of qmosRT-PCR assay using plasmid reference standards for quantification of nOPV2-c1_143 mutants, generated from the virus-spiked samples: (**A**) the mutant percentages generated by qmosRT-PCR are plotted against the expected percentages of mutants and (**B**) the mutant percentages generated by qmosRT-PCR are plotted against the results of the Illumina sequencing (NGS). The correlation line is drawn for the averages of the qmosRT-PCR results. The results of different qmosRT-PCR runs (Table 5) are presented with different colors.

Similarly, we spiked the mutant virus in the non-mutant virus; first, both viruses were titrated with a conventional CCID_50_ assay and the MPBT assay [25] and, as both methods generate similar results, the obtained titers were averaged and used to spike mutant virus in non-mutant virus. As the genome copy (GC) and CCID_50_ can differ, the GCs of the spiked viruses and the nOPV2 RNA references were determined by subjecting their stocks to quantitative RT-PCR using plasmids with known GCs as reference standards, as described in Section 2.5 above. The percentages of the spiked samples were recalculated based on the GCs of the virus stocks and they are presented in the first columns in Table 3, Table 4 and Table 5 (and in Supplement Tables S1–S3). Two sets of the same spiked samples were prepared and subjected to qmosRT-PCR assays using both plasmids and RNAs of mutants and non-mutants as reference standards. The results of the qmosRT-PCR assays using plasmids as reference standards are presented in Table 3, Table 4, Table 5 and Table 6 and in Figure 3, Figure 4 and Figure 5, and the results of the qmosRT-PCR using RNAs as reference standards are presented as supplements in Tables S1–S4 and Figures S1–S3. 

To quantify the levels of the VP1-N_171_D mutants, the RNA was extracted from the spiked samples and subjected to NGS and qmosRT-PCR assays. The generated results are presented in Table 3 (and in Supplement Table S1). These results showed that NGS generated similar results as the expected percentages prepared by spiking based on GCs. The qmosRT-PCR method was able to consistently detect mutations at levels above 1% (GC). The expected percentages of mutants based on GCs and those determined by NGS correlated very well with the percentages determined with the qmosRT-PCR method using plasmid standard curves, with R^2^ = 0.99 for both approaches (as shown in Figure 3A,B; see also the corresponding Supplementary Figure S1A,B, where R^2^ = 0.99).

For the quantification of the VP1-E_295_K mutants in nOPV2-c1, we spiked the mutant virus in non-mutant virus using CCID_50_ values; both viruses were titrated as mentioned above and used to spike mutants in non-mutants. Two sets of the same spiked samples were prepared on different days. As for the N171D mutants, fractions were also calculated based on the GC concentrations of the two stocks. Each spiked set of samples was subjected to three runs of the qmosRT-PCR assay on different days by the same operator. Each sample was analyzed in three replicates in each run. The two spiked sets were also subjected to Illumina sequencing as described above to confirm the percentages of the spiked samples and the results of the qmosRT-PCR. The results of the qmosRT-PCR and Illumina sequencing are summarized in Table 4 (and in the correspondent Supplementary Table S2). The qmosRT-PCR assay generated consistent results and was able to detect less than 1% of mutants in the spiked samples. Quantitatively similar results were obtained for spikes above 2% (GC). The expected percentages of mutants (GC) and those determined by NGS correlated well with percentages determined with the qmosRT-PCR method using GC units, with R^2^ = 1.00 and 0.99, respectively, as shown in Figure 4A,B (and in Supplementary Figure S2A,B, where R^2^ = 1:00).

To evaluate the quantification of VP1-I_143_T mutants in nOPV2-c1, we spiked the mutant virus into the non-mutant virus in a similar way as described above for the VP1-N_171_D mutants. The titrated viruses were used to spike mutants into non-mutants. As mentioned above, GC concentration fractions were calculated for subsequent use. Two sets of spiked samples with the same percentages of mutants were prepared on different days by the same operator and subjected to three runs of the qmosRT-PCR assay on different days; in total, six runs of qmosRT-PCR were performed for the two sets. Each sample was analyzed in three replicates in each run. The two sets of the spiked samples were also subjected to Illumina sequencing to confirm the percentages of mutants in the spiked samples and to compare them to the result of the qmosRT-PCR. The results of the qmosRT-PCR and Illumina sequencing are summarized in Table 5 (and in Supplementary Table S3). The qmosRT-PCR results include the mutants’ percentages calculated based on GC units. The qmosRT-PCR assay generated consistent results and was able to detect less than 1% of mutants in the spiked samples. Further, the expected percentages of mutants (GCs) and those determined by NGS correlated well with those determined with the qmosRT-PCR method with R^2^ = 0.97, as shown in Figure 5A,B (and in Figure S3A,B, where R^2^ = 0.95).

### 3.3. Evaluation of the Level of Mutant Variants in nOPV2 Vaccine Lots 

To evaluate the assays for the quantification of VP1-I_143_T, VP1-N_171_D, and VP1-E_295_K mutants in production stocks of nOPV2, different batches of the nOPV2 candidate 1 (nOPV2-c1) and candidate 2 (nOPV2-c2) vaccines with varying mutant percentages (manufactured by Bio Farma, Indonesia), as mentioned above in the Materials and Methods section, were tested by qmosRT-PCR and Illumina sequencing (NGS). Even though these assays were designed to specifically quantify the mutants in nOPV2-c1, nOPV2-c2 lots were tested in this experiment. The alignment of the primer and probe sequences to the nOPV2-c2 genome revealed the presence of one mismatch in the reverse primer (Pr143s-3011R) used for quantification of VP1-I143T mutants and two mismatches in the forward primer (nOPV2c1_3021F) used for quantification of VP1-N171D mutants. 

The NGS and qmosRT-PCR results using plasmids as reference standards are summarized in Table 6, and the results of the NGS and qmosRT-PCR using RNA as reference standards are summarized in Supplementary Table S4. The results in Table 6 show that the levels of VP1-I_143_T mutants were 0.00–1.93% and 0.00–1.60%, as detected by NGS and qmosRT-PCR, respectively. The levels of VP1-N_171_D mutants were 0.00–28.83% and 0.00–29.17%, as detected by NGS and qmosRT-PCR, respectively. The levels of VP1-E_295_K mutants were 2.07–45.83% and 0.08–62.49, as detected by NGS and qmosRT-PCR, respectively. In the nOPV2 lots tested, the nOPV2_295 mutants were detected at higher levels in comparison to the other mutants, followed by the nOPV2-171 mutants and then the VP1-I_143_T mutants, which were detected at a very low level by both PCR and NGS. 

**Table 6:** Quantification of nOPV2 mutants in nOPV2 batches by qmosRT-PCR assay using plasmids as reference standards.

nOPV2 Lots	nOPV2_295 Mutants, % ± SD			nOPV2_143 Mutants, % ± SD			nOPV2_171 Mutants, % ± SD		
	NGS	qmosRT-PCR	R^2^	NGS	qmosRT-PCR	R^2^	NGS	qmosRT-PCR	R^2^
nPOL2056C-c2	14.82 ± 0.89	27.49 ± 0.35	0.97	ND	ND	0.85	ND	ND	0.99
nPOL2018C	14.61 ± 0.52	23.98 ± 0.17		1.67 ± 0.08	1.44 ± 0.68		9.31 ± 0.27	7.71 ± 0.05	
nPOL2038C-c2	2.07 ± 0.13	0.08 ± 0.03		ND	ND		ND	0.02 ± 0.00	
nPOL2016C	45.83 ± 1.17	62.49 ± 2.16		0.21 ± 0.08	ND		0.9 ± 0.08	0.14 ± 0.08	
nPOL2B0419	32.71 ± 0.40	55.44 ± 1.02		1.19 ± 0.04	0.34 ± 0.16		24.18 ± 0.30	25.72 ± 0.72	
nPOL2B0519	37.92 ± 0.60	60.36 ± 1.24		1.11 ± 0.10	0.50 ± 0.24		28.83 ± 0.65	29.17 ± 3.48	
nPOL2-119C	4.97 ± 0.24	4.21 ± 0.69		0.99 ± 0.14	0.42 ± 0.37		3.7 ± 0.31	1.49 ± 0.36	
nPOL2-219C	7.37 ± 0.48	7.86 ± 0.77		1.93 ± 0.29	1.60 ± 0.30		5.26 ± 0.34	2.30 ± 0.79	
nPOL2-319C	7.35 ± 0.40	7.89 ± 0.57		1.63 ± 0.15	1.14 ± 0.52		5.23 ± 0.47	2.25 ± 0.65	

Note: NGS, next-generation sequencing (Illumina sequencing); SD, standard deviation; R^2^, coefficient of determination; ND, not detected.

Furthermore, the results (Table 6) show that there was a good correlation between the levels of VP1-N_171_D and VP1-E_295_K mutants determined by NGS and by the qmosRT-PCR assays, with R^2^ ≥ 0.97. The coefficient of correlation R^2^ observed between the levels of VP1-I_143_T mutants detected by NGS and by qmosRT-PCR assay was 0.85, and this was mostly due to the low frequency of VP1-I_143_T mutants. Similar results were generated by the qmosRT-PCR assay using viral RNAs as reference standards (Supplementary Table S4). The presence of the mismatches in the nOPV2 genome primers did not impact the quantification of the mutants in the nOPV2-c2 lots.

These results show that the qmosRT-PCR assay can be used to quantify levels of nOPV2 mutants as a part of quality control of nOPV2 lots. 

## 4. Discussion

Virus strains used in the manufacture of viral vaccines undergo mutational changes, which may cause reversion of attenuated strains to virulence, as well as changes in the antigenic properties that may affect the immunogenicity of vaccines [26,27,28]. Therefore, monitoring of the genetic consistency of vaccines is an important task to ensure their quality, safety, and potency.

Sabin strains used in the manufacture of OPV vaccine represent a case study of reversion of attenuated vaccine strains to virulence. Molecular studies performed in the 1980s showed that U_472_C mutation in Sabin type 3 virus [29], A_481_G mutation in Sabin type 2 virus [30], and G_480_A and U_525_C mutations in Sabin type 1 virus [31] accumulate during virus growth in vitro and in vivo and are responsible for reversion to virulence. Similar mutations are found in vaccine-derived strains isolated from cases of vaccine-associated paralytic polio and, therefore, the presence of these revertants in batches of polio vaccine is considered a safety risk. A method based on PCR and restriction enzyme cleavage (MAPREC) was developed to identify batches of oral polio vaccine (OPV) with unacceptable levels of neurovirulent revertants [32,33] and recommended by the WHO as an in vitro method of choice for lot release of OPV. However, MAPREC suffers from some shortcomings, including various technical challenges at multiple steps of its complex protocol.

Previously, we developed a method for quantification of mutants in Sabin 3 virus based on allele-specific primers that consist of three segments: the mutant-discriminating 3′-end, linked by a flexible polyinosine stretch to a specific segment that serves as an anchor to increase stability of binding and prevent non-specific priming [34]. Such a method needs the design of tethered allele-specific primers, extensive screening for the working primers, and optimization before it starts to work properly.

Quantitative PCR is an obvious alternative, but most PCR-based genotyping protocols quantify only one SNP allele per reaction [35,36,37,38,39,40].

nOPV2 was developed as a genetically stable vaccine to control outbreaks caused by cVDPV2. A recent study [41] comparing Sabin 2 virus and nOPV2 demonstrated significantly lower neurovirulence, as determined in a transgenic mouse assay, in the virus shed by recipients of nOPV2 compared to recipients of conventional OPV2. Furthermore, while shed Sabin 2 virus had the anticipated A481G reversion in the primary attenuation site in domain V in the 5′ UTR, associated with increased mouse neurovirulence, the stabilized domain V in the nOPV2 viruses did not show polymorphisms consistent with reversion to neurovirulence [41]. 

We previously reported on the use of NGS for quality control of nOPV2, with quantitative specifications imposed for the three mutations of interest studied in this work [21]. While NGS may be a viable approach for lot release, maintaining NGS assays and equipment in a validated state for indefinite routine release is, to our knowledge, unprecedented. As such, we sought to explore alternatives.

Quantitative PCR and NGS are broadly used to detect mutant variants in viruses and viral vaccines. Quantitative PCR has the advantage of being easy, faster, and cheaper to use to evaluate up to about four SNPs. NGS, in contrast, allows simultaneous analysis of many genomic loci; it is, however, more technically demanding and expensive. 

The method proposed in this communication is based on the use of two short minor groove binder probes; one specific for the mutants and the second specific for the non-mutants. Both are run in the same quantitative RT-PCR reaction with forward and reverse primers (Table 1), as described above. The results of the qmosRT-PCR, used to determine quantities of VP1 I_143_T, N_171_D, and E_295_K mutant variants that may affect the safety and immunogenicity of nOPV2, demonstrated a very good correlation with the results of the Illumina sequencing. This suggests that the qmosRT-PCR assay can be used for quantification of mutant variants in nOPV2 batches. Even though the method was designed to specifically quantify the mutants in the nOPV2-c1, the results for the quantification of the mutants in the nOPV2-c2 were comparable to those generated by NGS.

The method enables the quantification of both mutants and non-mutants in the same sample and in the same reaction, reducing time and labor and simplifying the method.

The proposed method is simple, sensitive, and could be used to test about 18 samples simultaneously, along with appropriate controls and reference samples. In addition, the use of different dyes to discriminate between mutants and non-mutants makes this method much easier to perform. The qmosRT-PCR method produces consistent results (with less than twofold difference from run to run) and is able to detect about 1% of mutant variants. 

The quantification of the mutant variants in nOPV2 lots correlated well with the results of the NGS, indicating that the qmosRT-PCR method could be used for quality control of the nOPV2 vaccine. 

In conclusion, the quantitative RT-PCR for quantification of undesirable mutants in the nOPV2 vaccine described in this communication offers a simple and rapid method for quantification of mutants in nOPV2 virus. The assays for quantitation of nOPV2 VP1-I_143_T, VP1-N_171_D, and VP1-E_295_K mutants were designed specifically to be applied for quality control during the manufacture of the nOPV2 vaccine.

## Figures and Tables

**Table 1:** Primers and probes used in qmosRT-PCR assay for quantification of the nOPV2-c1 VP1-143T, VP1-171D, and VP1-295K mutants.

Name of Oligos	Sequence 5′-->3′	Tm (Basic)	Oligo Size	Amplicon Size (bp)
Oligonucleotides for detection of VP1-I_143_T nOPV2-c1 mutant variants				
Pr143s-2938F (forward)	GGAGTTCACTTTTGTGGTCACCTC	56	24	74
Pr143s-3011R (reverse)	TCTGATAAACTTGGTTCAATGCATGTCCGT	59	30	
Prb143Cs12 FAM (mutant probe)	FAM-ACTACACTGATG–MGBNFQ	34	12	
Prb143Aa13 VIC (non-mutant probe)	VIC-GCATCAATGTAGT–MGBNFQ	36	13	
Oligonucleotides for detection of N_171_D nOPV2-c1 mutant variants				
nOPV2c1_3021F (forward)	TACCACCCGGAGCACCTATC	56	20	60
nOPV2c1_3080R (reverse)	TAGAGGACGTCTGCCACGTA	54	20	
Prb171G-a12FAM (mutant probe)	FAM-TCATCCCATTTA-MGBNFQ	36	12	
Prb171T-12VIC (non-mutant probe)	VIC-TCATTCCATTTA-MGBNFQ	30	12	
Oligonucleotides for detection of E_295_K nOPV2-c1 mutant variants				
Sab2_3396R (reverse)	GTGTCCAAATCCATAAGTCG	50	20	61
Sab2_3336F (forward)	TTATAAAGATGGGCTCACCC	50	20	
PrbA11VIC (mutant probe)	VIC-TACCAAAAAAG-MGBNFQ	28	11	
Prb295Ca13FAM (non-mutant probe)	FAM-CCTTTTCTGGTAG-MGBNFQ	38	13	

**Table 2:** Quantification of nOPV2-c1_171 mutants in plasmid-spiked samples by qmosRT-PCR assay using plasmids as reference standards.

Spiked Mutants %	Observed Mutants %	
	NGS	qmosRT-PCR
50.00	47.73	58.19 ± 2.22
25.00	24.06	28.53 ± 0.42
12.50	12.12	14.47 ± 1.84
6.30	6.17	6.01 ± 0.17
3.10	3.11	1.78 ± 0.22
1.60	2.07	0.41 ± 0.05
0.80	0.87	0.13 ± 0.02
0.40	0.42	0.01 ± 0.00
0.20	0.24	ND
0.10	0.13	ND

Note: NGS, Illumina sequencing; ND, not detected.

**Table 3:** Quantification of nOPV2-c1_171 mutants in the virus-spiked samples by qmosRT-PCR assay using plasmids as reference standards.

Expected Mutant % (GC #)	NGS (Set 1 and Set 2)	qmosRT-PCR Runs-Mutant %						
		Set 1 of Spiked Samples			Set 2 of Spiked Samples			Average ± SD
	Mutant % ± SD	Run 1	Run 2	Run 3	Run 4	Run 5	Run 6	
75.12	76.69 ± 0.27	89.68	81.88	80.04	89.98	89.42	89.96	86.83 ± 4.59
50.34	52.63 ± 2.25	68.42	53.02	54.33	69.74	68.30	69.08	63.81 ± 7.88
30.41	28.37 ± 0.59	41.17	31.03	32.49	49.39	47.39	49.81	41.88 ± 8.44
17.00	16.51 ± 1.08	22.07	13.31	14.46	28.88	27.00	32.27	23.00 ± 7.80
9.03	8.28 ± 0.02	10.25	6.94	7.63	14.37	13.09	15.99	11.38 ± 3.69
4.67	3.94 ± 0.26	2.56	2.36	2.27	5.47	4.86	7.49	4.17 ± 2.13
2.37	2.22 ± 0.35	0.28	0.55	0.42	1.24	1.07	1.68	0.87 ± 0.54
1.20	0.63 ± 0.21	0.01	0.12	0.07	0.16	0.13	0.34	0.14 ± 0.11
0.60	0.66 ± 0.29	ND	0.005	0.01	0.02	0.01	0.02	0.01 ± 0.01
0.30	0.42 ± 0.10	ND	ND	ND	ND	ND	ND	ND
0.15	ND	ND	ND	ND	ND	ND	ND	ND

Note: GC #, genome copy number; NGS, next-generation sequencing (Illumina sequencing); SD, standard deviation; ND, not detected.

**Table 4:** Quantification of nOPV2-c1_295 mutants in the virus-spiked samples by qmosRT-PCR assay using plasmids as reference standards.

Expected Mutant % (GC #)	NGS (Set 1 and Set 2)	qmosRT-PCR Runs						
		Set 1 of Spiked Samples			Set 2 of Spiked Samples			Average ± SD
	Mutant % ± SD	Run 1	Run 2	Run 3	Run 4	Run 5	Run 6	
53.29	39.28 ± 2.36	38.404	43.317	40.463	40.320	34.621	45.197	40.39 ± 3.71
27.60	16.00 ± 2.17	18.693	21.507	20.613	20.178	16.869	22.819	20.11 ± 2.10
14.06	7.89 ± 0.12	8.239	9.495	10.461	10.638	10.124	12.092	10.17 ± 1.28
7.09	3.56 ± 0.14	3.732	4.525	4.532	4.143	3.517	4.581	4.17 ± 0.46
3.56	1.45 ± 0.15	1.859	1.547	1.747	1.270	1.815	1.965	1.70 ± 0.25
1.79	0.76 ± 0.05	0.286	0.579	0.615	0.386	0.197	0.470	0.42 ± 0.16
0.89	0.46 ± 0.48	0.004	0.054	0.017	0.024	0.008	0.071	0.03 ± 0.03
0.45	ND	ND	ND	ND	ND	ND	ND	ND

Note: GC #, genome copy number; NGS, next-generation sequencing (Illumina sequencing); SD, standard deviation; ND, not detected.

**Table 5:** Quantification of nOPV2-c1_143 mutants in the virus-spiked samples with qmosRT-PCR assay using plasmids as reference standards.

Expected Mutant % (GC #)	NGS (Set 1 and Set 2)	qmosRT-PCR Runs—Mutants %						
		Set 1 of Spiked Samples			Set 2 of Spiked Samples			Average ± SD
	Mutant % ± SD	Run 1	Run 2	Run 3	Run 4	Run 5	Run 6	
40.42	60.98 ± 9.81	55.58	54.10	52.89	32.96	43.76	48.40	47.95 ± 8.52
18.68	29.03 ± 4.00	34.70	26.44	25.87	24.77	25.63	28.32	27.62 ± 3.67
9.02	13.48 ± 3.30	25.31	15.51	15.66	15.03	15.16	17.17	17.31 ± 4.00
4.44	6.52 ± 1.48	15.33	8.52	8.48	7.25	7.96	9.24	9.46 ± 2.95
2.20	3.42 ± 1.24	4.29	1.96	3.75	3.40	1.85	2.44	2.95 ± 1.01
1.10	1.59 ± 0.96	1.79	0.80	1.41	1.26	0.13	0.75	1.02 ± 0.59
0.55	0.94 ± 0.14	0.17	0.03	0.15	0.18	0.02	0.01	0.09 ± 0.08
0.27	2.38 ± 2.60	ND	ND	ND	ND	ND	ND	ND

Note: GC #, genome copy number; NGS, next-generation sequencing (Illumina sequencing); SD, standard deviation; ND, not detected.

## Data Availability

All relevant data are within the paper.

## Supplementary Materials

The following supporting information can be downloaded at: https://www.mdpi.com/article/10.3390/vaccines10091394/s1, Figure S1: Results of qmosRT-PCR assay using viral RNA reference standards for quantification of nOPV2-c1_171 mutants generated from the virus-spiked samples: (A) the mutant percentages generated by qmosRT-PCR are plotted against the expected percentages of mutants and (B) the mutant percentages generated by qmosRT-PCR are plotted against the results of Illumina sequencing (NGS). The correlation line is drawn for the averages of the qmosRT-PCR results. Figure S2: Results of qmosRT-PCR assay using viral RNA reference standards for quantification of nOPV2-c1_295 mutants generated from the virus-spiked samples: (A) the mutant percentages generated by qmosRT-PCR are plotted against the expected percentages of mutants and (B) the mutant percentages generated by qmosRT-PCR are plotted against the results of Illumina sequencing (NGS). The correlation line is drawn for the averages of the qmosRT-PCR results. Figure S3: Results of qmosRT-PCR assay using viral RNA reference standards for quantification of nOPV2-c1_143 mutants generated from the virus-spiked samples: (A) the mutant percentages generated by qmosRT-PCR are plotted against the expected percentages of mutants and (B) the mutant percentages generated by qmosRT-PCR are plotted against the results of Illumina sequencing (NGS). The correlation line is drawn for the averages of the qmosRT-PCR results. Table S1: Quantification of nOPV2-c1_171 mutants in the virus-spiked samples by qmosRT-PCR assay using viral RNA as reference standards. Table S2: Quantification of nOPV2-c1_295 mutants in the virus-spiked samples by qmosRT-PCR assay using viral RNAs as reference standards. Table S3: Quantification of nOPV2-c1_143 mutants in the virus-spiked samples by QmosRT-PCR assay using viral RNAs as reference standards. TableS4: Quantification of nOPV2 mutants in nOPV2 batches by qmosRT-PCR assay using viral RNA as reference standards.

## References

[1] Sutter R.W., Kew O.M., Cochi S.L., Aylward R.B., Plotkin S.A., Orenstein W.A., Offit P.A. (1993). Poliovirus vaccine—Live. Vaccines.

[2] John J., Giri S., Karthikeyan A.S., Iturriza-Gomara M., Muliyil J., Abraham A., Grassly N.C., Kang G. (2014). Effect of a single inactivated poliovirus vaccine dose on intestinal immunity against poliovirus in children previously given oral vaccine: An open-label, randomised controlled trial. Lancet.

[3] Rubin J., Ottosen A., Ghazieh A., Fournier-Caruana J., Ntow A.K., Gonzalez A.R. (2017). Managing the Planned Cessation of a Global Supply Market: Lessons Learned from the Global Cessation of the Trivalent Oral Poliovirus Vaccine Market. J. Infect. Dis..

[4] Brickley E.B., Strauch C.B., Wieland-Alter W.F., Connor R.I., Lin S., Weiner J.A., Ackerman M.E., Arita M., Oberste M.S., Weldon W.C. (2018). Intestinal Immune Responses to Type 2 Oral Polio Vaccine (OPV) Challenge in Infants Previously Immunized with Bivalent OPV and Either High-Dose or Standard Inactivated Polio Vaccine. J. Infect. Dis..

[5] Thompson K.M., Duintjer Tebbens R.J. (2017). Lessons from the Polio Endgame: Overcoming the Failure to Vaccinate and the Role of Subpopulations in Maintaining Transmission. J. Infect. Dis..

[6] Wright P.F., Connor R.I., Wieland-Alter W.F., Hoen A.G., Boesch A.W., Ackerman M.E., Oberste M.S., Gast C., Brickley E.B., Asturias E.J. (2016). Vaccine-induced mucosal immunity to poliovirus: Analysis of cohorts from an open-label, randomised controlled trial in Latin American infants. Lancet Infect. Dis..

[7] Wang H. (2020). Why Have cVDPV2 Outbreaks Increased Globally after the Polio Immunization Strategy Switch: Challenges for the Polio Eradication Endgame. China CDC Wkly..

[8] Blake I.M., Pons-Salort M., Molodecky N.A., Diop O.M., Chenoweth P., Bandyopadhyay A.S., Zaffran M., Sutter R.W., Grassly N.C. (2018). Type 2 Poliovirus Detection after Global withdrawal of Trivalent Oral Vaccine. N. Engl. J. Med..

[9] Jorba J., Diop O.M., Iber J., Henderson E., Zhao K., Quddus A., Sutter R., Vertefeuille J.F., Wenger J., Wassilak S.G.F. (2019). Update on Vaccine-Derived Poliovirus Outbreaks—Worldwide, January 2018–June 2019. MMWR Morb. Mortal. Wkly. Rep..

[10] Evans D.M., Dunn G., Minor P.D., Schild G.C., Cann A.J., Stanway G., Almond J.W., Currey K., Maizel J.V. (1985). Increased neurovirulence associated with a single nucleotide change in a noncoding region of the Sabin type 3 poliovaccine genome. Nature.

[11] Macadam A.J., Ferguson G., Burlison J., Stone D., Skuce R., Almond J.W., Minor P.D. (1992). Correlation of RNA secondary structure and attenuation of Sabin vaccine strains of poliovirus in tissue culture. Virology.

[12] Macadam A.J., Stone D.M., Almond J.W., Minor P.D. (1994). The 5′ noncoding region and virulence of poliovirus vaccine strains. Trends Microbiol..

[13] Stern A., Yeh M.T., Zinger T., Smith M., Wright C., Ling G., Nielsen R., Macadam A., Andino R. (2017). The Evolutionary Pathway to Virulence of an RNA Virus. Cell.

[14] Dunn G., Begg N.T., Cammack N., Minor P.D. (1990). Virus excretion and mutation by infants following primary vaccination with live oral poliovaccine from two sources. J. Med. Virol..

[15] Laassri M., Dragunsky E., Enterline J., Eremeeva T., Ivanova O., Lottenbach K., Belshe R., Chumakov K. (2005). Genomic analysis of vaccine-derived poliovirus strains in stool specimens by combination of full-length PCR and oligonucleotide microarray hybridization. J. Clin. Microbiol..

[16] Laassri M., Lottenbach K., Belshe R., Rennels M., Plotkin S., Chumakov K. (2006). Analysis of reversions in the 5′-untranslated region of attenuated poliovirus after sequential administration of inactivated and oral poliovirus vaccines. J. Infect. Dis..

[17] Konopka-Anstadt J.L., Campagnoli R., Vincent A., Shaw J., Wei L., Wynn N.T., Smithee S.E., Bujaki E., Te Yeh M., Laassri M. (2020). Development of a new oral poliovirus vaccine for the eradication end game using codon deoptimization. NPJ Vaccines.

[18] Yeh M.T., Bujaki E., Dolan P.T., Smith M., Wahid R., Konz J., Weiner A.J., Bandyopadhyay A.S., Van Damme P., De Coster I. (2020). Engineering the Live-Attenuated Polio Vaccine to Prevent Reversion to Virulence. Cell Host Microbe.

[19] Macadam A.J., Ferguson G., Stone D.M., Meredith J., Knowlson S., Auda G., Almond J.W., Minor P.D. (2006). Rational design of genetically stable, live-attenuated poliovirus vaccines of all three serotypes: Relevance to poliomyelitis eradication. J. Virol..

[20] WHO (2021). WHO: Meeting of Strategic Advisory Group of Experts on Immunization, October 2021: Conclusions and Recommendations.

[21] Konz J.O., Schofield T., Carlyle S., Wahid R., Ansari A., Strating J., Yeh M.T., Manukyan H., Smits S.L., Tritama E. (2021). Evaluation and validation of next-generation sequencing to support lot release for a novel type 2 oral poliovirus vaccine. Vaccine X.

[22] Van Damme P., De Coster I., Revets H., Bandyopadhyay A.S. (2019). Poliopolis. Lancet.

[23] Simonyan V., Mazumder R. (2014). High-Performance Integrated Virtual Environment (HIVE) Tools and Applications for Big Data Analysis. Genes.

[24] Laassri M., Cherkasova E., Abu-Asab M.S., Chumakov K., Garcia M. (2012). Microarray Techniques for Evaluation of Genetic Stability of Live Viral Vaccines. Viral Genomes—Molecular Structure, Diversity, Gene Expression Mechanisms and Host-Virus Interactions.

[25] Manukyan H., Rodionova E., Zagorodnyaya T., Lin T.L., Chumakov K., Laassri M. (2019). Multiplex PCR-based titration (MPBT) assay for determination of infectious titers of the three Sabin strains of live poliovirus vaccine. Virol. J..

[26] Gambaryan A.S., Marinina V.P., Tuzikov A.B., Bovin N.V., Rudneva I.A., Sinitsyn B.V., Shilov A.A., Matrosovich M.N. (1998). Effects of host-dependent glycosylation of hemagglutinin on receptor-binding properties on H1N1 human influenza A virus grown in MDCK cells and in embryonated eggs. Virology.

[27] Hughes M.T., McGregor M., Suzuki T., Suzuki Y., Kawaoka Y. (2001). Adaptation of influenza A viruses to cells expressing low levels of sialic acid leads to loss of neuraminidase activity. J. Virol..

[28] Schild G.C., Oxford J.S., de Jong J.C., Webster R.G. (1983). Evidence for host-cell selection of influenza virus antigenic variants. Nature.

[29] Cann A.J., Stanway G., Hughes P.J., Minor P.D., Evans D.M., Schild G.C., Almond J.W. (1984). Reversion to neurovirulence of the live-attenuated Sabin type 3 oral poliovirus vaccine. Nucleic Acids Res..

[30] Macadam A.J., Pollard S.R., Ferguson G., Skuce R., Wood D., Almond J.W., Minor P.D. (1993). Genetic basis of attenuation of the Sabin type 2 vaccine strain of poliovirus in primates. Virology.

[31] Christodoulou C., Colbere-Garapin F., Macadam A., Taffs L.F., Marsden S., Minor P., Horaud F. (1990). Mapping of mutations associated with neurovirulence in monkeys infected with Sabin 1 poliovirus revertants selected at high temperature. J. Virol..

[32] Chumakov K.M. (1999). Molecular consistency monitoring of oral poliovirus vaccine and other live viral vaccines. Dev. Biol. Stand..

[33] Chumakov K.M., Powers L.B., Noonan K.E., Roninson I.B., Levenbook I.S. (1991). Correlation between amount of virus with altered nucleotide sequence and the monkey test for acceptability of oral poliovirus vaccine. Proc. Natl. Acad. Sci. USA.

[34] Bidzhieva B., Laassri M., Chumakov K. (2014). Allele-specific PCR for quantitative analysis of mutants in live viral vaccines. J. Virol. Methods.

[35] Delobel P., Saliou A., Nicot F., Dubois M., Trancart S., Tangre P., Aboulker J.P., Taburet A.M., Molina J.M., Massip P. (2011). Minor HIV-1 variants with the K103N resistance mutation during intermittent efavirenz-containing antiretroviral therapy and virological failure. PLoS ONE.

[36] Germer S., Holland M.J., Higuchi R. (2000). High-throughput SNP allele-frequency determination in pooled DNA samples by kinetic PCR. Genome Res..

[37] Kianianmomeni A., Schwarz G., Felsenstein F.G., Wenzel G. (2007). Validation of a real-time PCR for the quantitative estimation of a G143A mutation in the cytochrome bc1 gene of Pyrenophora teres. Pest Manag. Sci..

[38] Punia P., Cane P., Teo C.G., Saunders N. (2004). Quantitation of hepatitis B lamivudine resistant mutants by real-time amplification refractory mutation system PCR. J. Hepatol..

[39] Schwarz G., Baumler S., Block A., Felsenstein F.G., Wenzel G. (2004). Determination of detection and quantification limits for SNP allele frequency estimation in DNA pools using real time PCR. Nucleic Acids Res..

[40] Taira C., Matsuda K., Saito S., Sakashita K., Sugano M., Okumura N., Honda T. (2012). Application of allele-specific quantitative PCR using genomic DNA to monitor minimal residual disease based on mutant gene levels following allogeneic hematopoietic stem cell transplantation in patients with hematological malignancies: Comparison of mutant levels with autologous DNA percentage by short tandem repeat-PCR. Clin. Chim. Acta.

[41] Wahid R., Mercer L., Gast C., De Leon T., Saez-Llorens X., Fix A., Macadam A., Stephens L., Chumakov K., Smits S.L. (2022). Evaluating stability of attenuated Sabin and two novel type 2 oral poliovirus vaccines in children. NPJ Vaccines.

