# Supplementary material for: Quantitative RT-PCR Assays for Quantification of Undesirable Mutants in the Novel Type 2 Oral Poliovirus Vaccine
Source: Vaccines (Basel). 2022 Aug 25;10(9):1394. doi: 10.3390/vaccines10091394 (PMC9502871; doi:10.3390/vaccines10091394)
Supplement: Supplementary file 1 [file vaccines-10-01394-s001.zip › vaccines-1853173-SM.pdf]

vaccines

Supplementary Materials

Table S1: Quantification of nOPV2-c1_171 mutants in the virus-spiked samples by qmosRT-PCR assay using viral RNA as reference standards

Expected Mutant % (GC #)	NGS (set 1 and set 2)	qmosRT-PCR runs- mutant %						
		Set 1 of spiked samples			Set 2 of spiked samples			
	mutant % ± SD	Run 1	Run 2	Run3	Run 4	Run 5	Run 6	Average ± SD
75.12	76.69 ± 0.27	88.33	85.53	84.52	88.16	84.86	85.27	86.11 ± 1.69
50.34	52.63 ± 2.25	66.31	62.14	65.11	65.19	60.00	59.93	63.11 ± 2.80
30.41	28.37 ± 0.59	38.26	41.23	45.78	43.53	39.46	40.28	41.42 ± 2.78
17.00	16.51 ± 1.08	18.96	20.59	24.64	23.76	21.78	24.72	22.41 ± 2.36
9.03	8.28 ± 0.02	8.84	11.68	14.70	11.08	10.56	11.78	11.44 ± 1.92
4.67	3.94 ± 0.26	2.14	4.47	5.30	3.98	4.04	5.45	4.23 ± 1.20
2.37	2.22 ± 0.35	0.22	1.18	1.22	0.84	0.95	1.24	0.94 ± 0.39
1.20	0.63 ± 0.21	0.010	0.29	0.22	0.10	0.13	0.25	0.17 ± 0.10
0.60	0.66 ± 0.29	ND	0.02	0.02	0.01	0.02	0.02	0.02 ± 0.004
0.30	0.42 ± 0.10	ND	ND	ND	ND	ND	ND	ND

Note: GC #; Genome copy number, NGS; New generation of sequencing (Illumina sequencing), SD; Standard deviation error, ND; Not detected

Table S2: Quantification of nOPV2-c1_295 mutants in the virus-spiked samples by qmosRT-PCR assay using viral RNAs as reference standards

Expected Mutant % (GC #)	NGS (set1 and set 2)	qmosRT-PCR runs, mutant %						
		Set 1 of spiked samples			Set 2 of spiked samples			
	mutant % ± SD	Run 1	Run 2	Run3	Run 4	Run 5	Run 6	Average ± SD
53.29	39.28 ± 2.36	32.122	43.334	34.285	28.127	28.562	35.968	33.73 ± 5.63
27.60	16.00 ± 2.17	15.122	21.705	16.306	12.383	12.509	16.332	15.72 ± 3.42
14.06	7.89 ± 0.12	6.614	9.666	7.719	6.072	6.945	7.867	7.48 ± 1.27
7.09	3.56 ± 0.14	3.014	4.644	3.140	2.209	2.107	2.620	2.96 ± 0.92
3.56	1.45 ± 0.15	1.517	1.604	1.158	0.627	1.047	1.103	1.18 ± 0.35
1.79	0.76 ± 0.05	0.241	0.606	0.380	0.178	0.083	0.228	0.29 ± 0.18
0.89	0.46 ± 0.48	0.004	0.058	0.009	0.010	0.003	0.033	0.02 ± 0.02
0.45	ND	ND	ND	ND	ND	ND	ND	ND

Note: GC #; Genome copy number, NGS; New generation of sequencing (Illumina sequencing), SD; Standard deviation, ND; Not detected

vaccines

MDPI

Table S3: Quantification of nOPV2-c1_143 mutants in the virus-spiked samples by QmosRT-PCR assay using viral RNAs as reference standards

Expected Mutant % (GC#)	NGS (set1 and set 2)	qmosRT-PCR runs - mutants %						
		Set 1 of spiked samples			Set 2 of spiked samples			
	mutant % ± SD	Run 1	Run 2	Run3	Run 4	Run 5	Run 6	Average ± SD
40.42	60.98 ± 9.81	64.16	61.38	56.64	38.77	41.66	46.49	51.52 ± 10.66
18.68	29.03 ± 4.00	43.68	34.22	29.69	30.31	23.82	27.66	31.56 ± 6.84
9.02	13.48 ± 3.30	33.68	22.13	18.44	19.14	14.06	17.19	20.77 ± 6.85
4.44	6.52 ± 1.48	21.57	12.94	10.35	9.33	7.50	9.49	11.86 ± 5.07
2.20	3.42 ± 1.24	6.59	3.43	4.73	4.53	1.74	2.70	3.95 ± 1.71
1.10	1.59 ± 0.96	2.92	1.52	1.82	1.71	0.12	0.88	1.50 ± 0.94
0.55	0.94 ± 0.14	0.30	0.08	0.20	0.25	0.02	0.02	0.14 ± 0.12
0.27	2.38 ± 2.60	ND	ND	ND	ND	ND	ND	ND

Note: GC#; Genome copy number, NGS; New generation of sequencing (Illumina sequencing), SD; Standard deviation, ND; Not detected

Table S4: Quantification of nOPV2 mutants in nOPV2 batches by qmosRT-PCR assay using viral RNA as reference standards

nOPV2 lots	nOPV2_295 mutants, % ± SD			nOPV2_143 mutants, % ± SD			nOPV2_171 mutants, % ± SD		
	NGS	qmosRT-PCR	R^2	NGS	qmosRT-PCR	R^2	NGS	qmosRT-PCR	R^2
nPOL2056C-c2	14.82 ± 0.89	22.79 ± 0.24	0.99	ND	ND	0.85	ND	ND	0.99
nPOL2018C	14.61 ± 0.52	21.29 ± 0.10		1.67 ± 0.08	1.15 ± 0.51		9.31 ± 0.27	8.03 ± 0.44	
nPOL2038C-c2	2.07 ± 0.13	0.07 ± 0.03		ND	ND		ND	0.02 ± 0.00	
nPOL2016C	45.83 ± 1.17	60.25 ± 2.12		0.21 ± 0.08	ND		0.9 ± 0.08	0.17 ± 0.08	
nPOL2B0419	32.71 ± 0.40	48.35 ± 1.36		1.19 ± 0.04	0.28 ± 0.12		24.18 ± 0.30	24.91 ± 0.72	
nPOL2B0519	37.92 ± 0.60	53.42 ± 1.38		1.11 ± 0.10	0.40 ± 0.18		28.83 ± 0.65	28.11 ± 3.22	
nPOL2-119C	4.97 ± 0.24	4.20 ± 0.64		0.99 ± 0.14	0.38 ± 0.31		3.7 ± 0.31	1.75 ± 0.64	
nPOL2-219C	7.37 ± 0.48	7.70 ± 0.70		1.93 ± 0.29	1.33 ± 0.23		5.26 ± 0.34	2.64 ± 1.17	
nPOL2-319C	7.35 ± 0.40	7.31 ± 0.44		1.63 ± 0.15	0.95 ± 0.41		5.23 ± 0.47	2.53 ± 0.93	

Note: NGS; Next-generation sequencing (Illumina sequencing), SD; Standard deviation, R2: Coefficient of determination, ND; Not detected

vaccines

MDPI

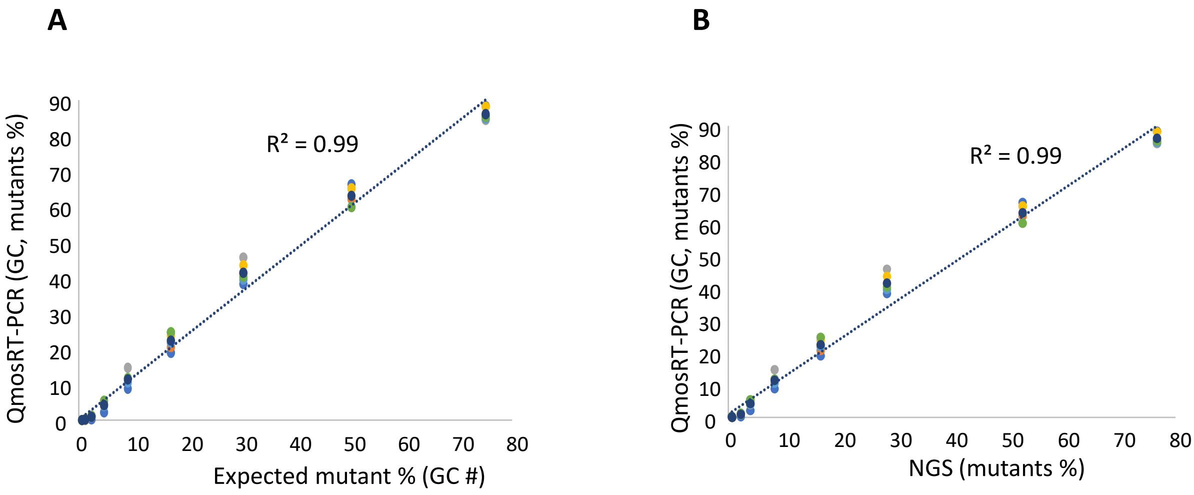

Fig. S1: Results of qmosRT-PCR assay using viral RNA reference standards for quantification of nOPV2-c1_171 mutants, generated from the virus-spiked samples: **A**; the mutant percentages generated by qmosRT-PCR are plotted against the expected percentages of mutants and **B**; the mutant percentages generated by qmosRT-PCR are plotted against the results of Illumina sequencing (NGS). The correlation line is drawn for the averages of the qmosRT-PCR results. The results of different qmosRT-PCR runs (Table S1) are presented with different colors.

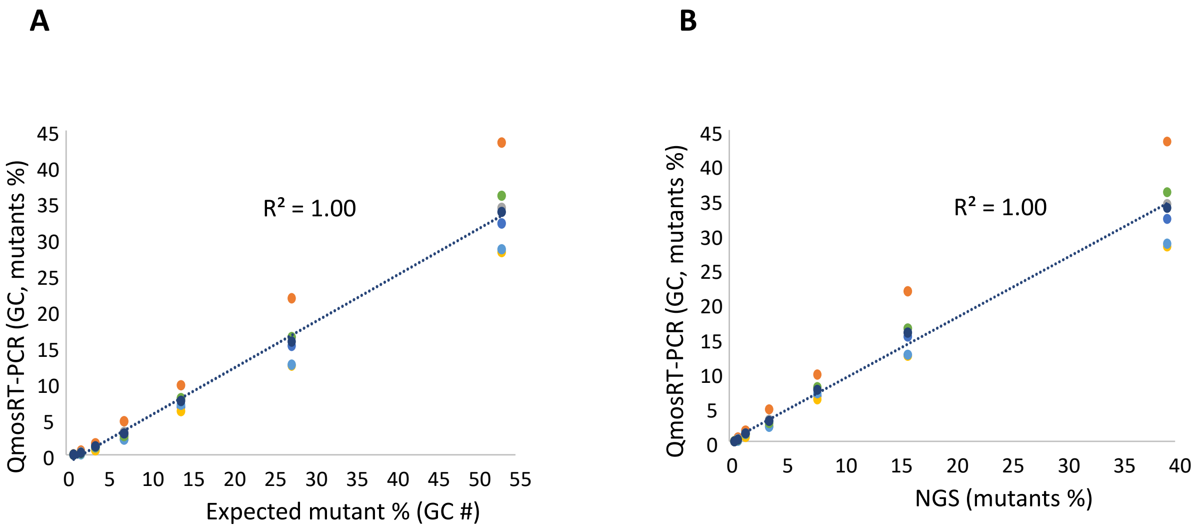

vaccines

Fig. S2: Results of qmosRT-PCR assay using viral RNA reference standards for quantification of nOPV2-c1_295 mutants, generated from the virus-spiked samples: **A**; the mutant percentages generated by qmosRT-PCR are plotted against the expected percentages of mutants and **B**; the mutant percentages generated by qmosRT-PCR are plotted against the results of Illumina sequencing (NGS). The correlation line is drawn for the averages of the qmosRT-PCR results. The results of different qmosRT-PCR runs (Table S2) are presented with different colors.

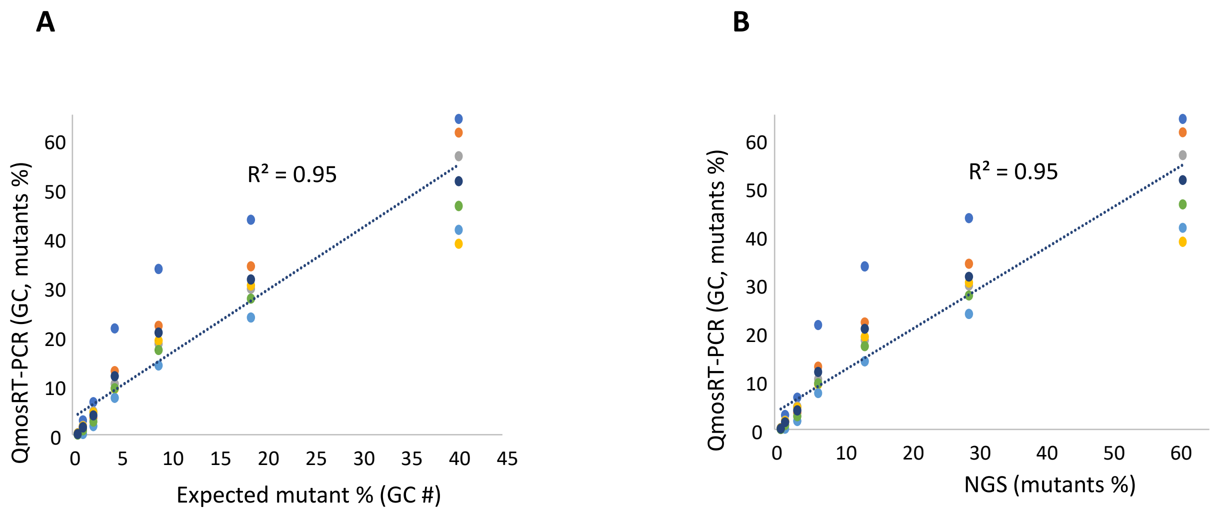

Fig. S3: Results of qmosRT-PCR assay using viral RNA reference standards for quantification of nOPV2-c1_143 mutants, generated from the virus-spiked samples: **A**; the mutant percentages generated by qmosRT-PCR are plotted against the expected percentages of mutants and **B**; the mutant percentages generated by qmosRT-PCR are plotted against the results of Illumina sequencing (NGS). The correlation line is drawn for the averages of the qmosRT-PCR results. The results of different qmosRT-PCR runs (Table S3) are presented with different colors.
